# Efficient Catalysis by Sodium Hypophosphite for Solid-State Polymerization of High-Viscosity PA6/66 Copolyamide: Kinetics, Process Optimization, and Industrial Application

**DOI:** 10.3390/polym18030384

**Published:** 2026-01-31

**Authors:** Feng Jiang, Chunxiao Yu, Zhiyu Hu, Yilan Wu, Xin Li

**Affiliations:** 1 State Key Laboratory of Advanced Fiber Materials, College of Material Science and Engineering, Donghua University, Shanghai 201620, China; 2 State Key Laboratory of Bio-Based Fiber Materials, China Textile Academy, Beijing 100025, China; 3 China General Technology (Group) Holding Co., Ltd., Beijing 100055, China

**Keywords:** PA6/66 copolyamide, solid state polymerization, catalytic reaction, control concentration, reaction kinetics

## Abstract

The industrial preparation via solid-state polymerization (SSP) of high-viscosity copolyamides 6/66 (PA6/66) addresses the challenges, including prolonged reaction times, high energy consumption, and uneven viscosity distribution. In this study, sodium hypophosphite was introduced into the PA6/66 copolymerization system as a solid-state polymerization catalyst. The effects of this catalyst on the solid-state viscosity-increasing rate and relative viscosity were systematically investigated, and the extraction process was optimized to solve the loss of catalyst and controllable extractable content. The results showed that the relative viscosity of PA6/66 increased linearly with the SSP time, and the apparent viscosity increase rate could be stably maintained at 0.14 h^−1^ at 160 °C due to the catalytic action. Based on the phosphorus (P) content in the chips, the viscosity increase rate is not further large when the P content is 25 ppm at 150 °C and 30 ppm at 160 °C, which can be added as a “control concentration” as a catalyst. The extraction kinetics showed that the catalyst concentration of the chip could be kept higher than the control concentration, and the extractable content can satisfy the requirements for processing. The catalyst of sodium hypophosphite was utilized on the 4500 tons/year PA6/66 continuous polymerization test line, and the high-viscosity PA6/66 chips with uniform viscosity were stably prepared. This study provides a reliable theoretical basis and process route for the large-scale continuous preparation of high-quality and high-viscosity PA6/66 resin.

## 1. Introduction

Copolyamide 6/66 (PA6/66) has the characteristics of low melting point, easy dyeing, softness, and good transparency, which is an important polyamide 6 (PA6) copolymer-modified product that has attracted much attention in recent years [1,2]. Copolyamide 6/66 with high relative viscosity (≥4.0) has higher melt strength, higher mechanical properties, and barrier properties, and is an excellent raw material for multi-layer composite films, high-strength industrial filaments, and industrial filter felts.

Several methods have been developed to fabricate high-relative-viscosity polyamide, including chemical chain extension, extension of melt polymerization time, and solid-state polymerization [3,4,5,6]. Solid-state polymerization (SSP) involves heating the starting material in an oxygen-free atmosphere (i.e., under flowing gas or high pressure or in a vacuum) at a temperature below the melting point, increasing the molecular weight of the product while the material retains its solid shape. Its advantages over conventional melt-phase operations are the low polymerization temperatures, eliminating decomposition and undesirable by-product formation, and the simplicity and low cost of process equipment. The method can be used for continuous industrial production and is the main method for preparing high-relative-viscosity polyamide products in the industry [7,8].

The research on solid-state polymerization focuses on the physical and chemical reaction processes in the SSP process. Zimmerman J. [9] studied that the solid-phase polymerization reaction mainly occurs in the amorphous region of the polymer. The active end groups in the amorphous region can be close to each other by diffusion. When the distance between them is close enough, the polymerization reaction can occur. Chen F.C. et al. [10] found that the reaction temperature is the key factor affecting the reaction rate of SSP. In the solid-state polymerization reaction, the chemical reaction rate and the diffusion rate of the generated small molecular by-products jointly affect the rate of the solid-state polymerization reaction. When the reaction temperature changes, the influence of the two factors will be different. Generally, the reaction temperature of SSP should be between 20 °C and 160 °C below the melting point of a prepolymer [11]. Gaymans et al. [8] also found that the viscosity-increasing rate of the product increased first and then decreased with the extension of reaction time when they studied the SSP of nylon 6 and nylon 46. The relative viscosity of the product increased with the extension of reaction time. To increase the reaction rate of SSP, a catalyst can be added. The catalysts reported in the literature for SSP of polyamides include acids (H_2_SO_4_, H_3_PO_4_, H_3_BO_3_, etc.), metal oxides (MgO, SbF_3_, As_2_O_3_, etc.), phosphorus-containing polymers, etc. [8,12,13,14,15,16]. There are few reports on the dispersion methods of catalysts in prepolymers or monomer salts and their effects on SSP. Papaspyrides [17] studied the solid-phase polymerization of sulfuric acid, phosphoric acid, and boric acid catalysts after co-precipitation with diammonium dodecylmethylene adipate nylon salt. Compared with the previous catalyst and nylon salt or prepolymer blending method, the obtained catalyst is highly dispersed in the raw material, and the SSP reaction rate is faster. About the SSP influencing factors and kinetics of polyamide, some literature [18,19,20,21,22,23] reported the effects of amorphous structure, amorphous content, molecular weight, and end-group concentration of the polymer on the reaction rate and studied the relevant kinetic model. The above studies mainly focused on the SSP of nylon 6 and nylon 66. The effects of PA6/66 catalytic solid-state polymerization, extremely low concentration catalyst content, and catalyst dispersion on the viscosity uniformity of chips have not been reported in the literature.

In this paper, a highly active catalyst (sodium hypophosphite) for solid-state polymerization was found. By adding it to the copolymerization of PA6/66, high-viscosity resin chips were obtained by extraction, drying, and solid-state polymerization. The effect of the catalyst on the viscosity of the chip was studied, and the optimal control concentration of the solid-state polymerization reaction was determined. The problem of the inconsistency between the inner and outer layers of the viscosity of the high-viscosity chip caused by the uneven distribution of the catalytically active component in the chip was solved by optimizing the extraction process. This study provides important theoretical support and technical reference for the optimization of the preparation process of high-viscosity polyamide chips.

## 2. Materials and Methods

### 2.1. Materials

Polymerization grade ε-caprolactam (CPL,99%) was purchased from Zhejiang Juhua Co., Ltd. (Hangzhou, China). Nylon 66 salt and sodium hypophosphite (≥99.5) were purchased from Macklin Biochemical Technology Co., Ltd. (Shanghai, China). All chemicals were used without any purification, and the water used was deionized water in this investigation.

### 2.2. Synthesis of PA6/66 Copolyamides

The polymerization process of PA6/66 copolyamides mainly includes the hydrolytic ring-opening polymerization (ROP) and melt polycondensation of PA6 with the PA66 salt (15 wt.%). Sodium hypophosphite, CPL, and PA66 salt (containing 2 wt.% DI water as a ring opener) were polymerized in a 30 L high-pressure reactor (Huitong Technology Co. Ltd. Yangzhou, China) equipped with a central mechanical stirrer, a nitrogen gas inlet and outlet, and a vacuum system. The mass percentage of sodium hypophosphite added to the solid feeding amount was 250 ppm−1300 ppm. The oxygen of the closed reactor was removed by nitrogen to avoid the polymer’s oxidation. The polymerization temperature was maintained at 240 °C for 3 h (polymerization pressure: 0.4 MPa), then the internal pressure of the reactor was gradually released to atmospheric pressure, and the reaction temperature was gradually heated to 250 °C for 4 h. In the final reaction stage, the flowing nitrogen atmosphere was maintained to exhaust water from the condensation polymerization until the torque value (i.e., melting viscosity) reached a target quantity that ensured a sufficient degree of polymerization of the polymer. The copolymers were extruded from the reactor, cooled, pelletized, and extracted with superheated water. The prepared cylindrical chip had a diameter of 2 mm and a length of 2 mm, with a relative viscosity of 2.65–2.71. After vacuum drying at 95 °C for 24 h, the chips were analyzed and tested.

### 2.3. Solid-State Polymerization of PA6/66 Copolyamides

In order to obtain high-viscosity PA6/66 resin, solid-state polymerization is usually used. The prepolymer chips, prepared in Section 2.2, were added to the reactor (China Textile Academy, Beijing, China), and the air was replaced by nitrogen (N_2_) for 1 h. The reaction temperature was maintained at a constant value (150, 155, and 160 °C), and the temperature fluctuation was less than 1 °C. The mass flow meter was used to control the N_2_ to purge the solid-state polymerization reactor at a constant speed of 30 mL/min. After the reaction time was reached, the chips were taken out after cooling to below 100 °C.

### 2.4. Characterization

#### 2.4.1. Relative Viscosity Analysis

The relative viscosity test was carried out according to the GB/T 38138-2019 [24]. The chip (0.5000 g) was placed in a conical flask, and 50 mL of concentrated sulfuric acid (concentration of 96%) was added to the conical flask with a pipette. After sealing the mouth of the bottle with a sealing film, the conical bottle was shaken and dissolved in a constant temperature water bath at 40 °C. The relative viscosity of the sample was tested using an IVS300-4 intelligent viscosity measuring instrument (Hangzhou Zhongwang Technology Co., Ltd., Hangzhou, China). The diameter of the test unit viscometer was 1.08 mm, and the test temperature was 25 °C. The intelligent viscosity measuring instrument can automatically read the relative viscosity value of the sample and take the average value after 5 parallel tests to obtain the relative viscosity value of the sample.

#### 2.4.2. Hot Water Extractable Substance Analysis

The hot water extractable substance test was carried out according to the GB/T 38138-2019 [24]. First, 20.000 g of the sample was accurately weighed (with a precision of 0.1 mg) and transferred to a Sox let extractor. The extraction was performed at a temperature higher than 98 °C for 12 h. After the extraction was completed, the extract was prepared into a 200 mL solution. Finally, the refractive index of the three extracts was measured in parallel at 25 °C, and the average value was taken as the final refractive index value. By measuring the refractive index of the extract, the extractable content was calculated according to Equations (1) and (2).

(1) X=(Y−B)/A

(2) $$P\%=\frac{X*200}{M}*100\%$$

where *X* represents the concentration of the extraction solution (mg/mL), *Y* represents the refractive coefficient of the extract solution, *M* represents the weight of the sample transferred to the Sox let extractor (g), *P* represents the hot water extractable substances (%), *A* = 1.51259 × 10^−4^, and *B* = 1.33247.

#### 2.4.3. Element Content Analysis

Agilent ICPOES730 (Agilent Technologies, Inc., Santa Clara, CA, USA) was used to analyze the element content of the chips. The carrier gas was argon, the plasma gas flow rate was 1.5 L/min, and the detection mode was axial observation. The content of the P element in the chips was obtained.

## 3. Results and Discussion

### 3.1. The Effect of Catalyst on SSP of PA6/66

**Figure 1:**
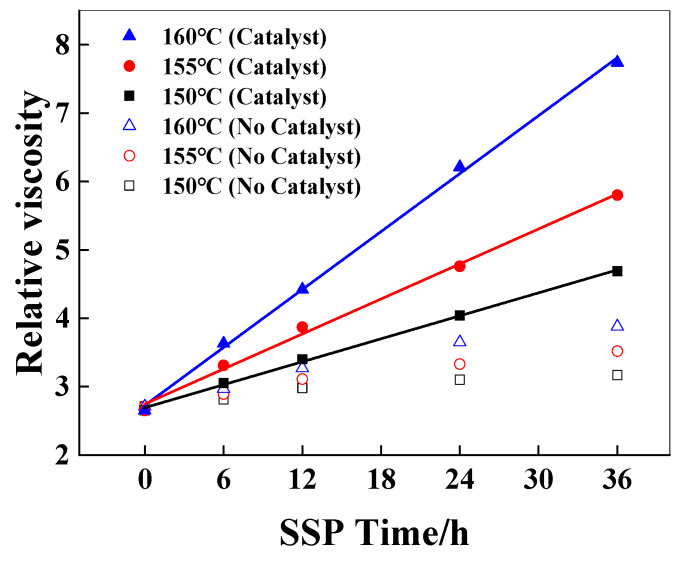
The effect of the catalyst on the relative viscosity of PA6/66 chips.

Sodium hypophosphite is a common additive, which was added in the melt polymerization of polyamide 66 to reduce the activation energy of the polycondensation reaction between the terminal carboxyl group and the terminal amino group [25], and plays an anti-yellowing role in polymerization and subsequent processing [26]. The solid-state polymerization of PA6/66 chips with a relative viscosity of 2.65 with a catalyst and 2.71 without a catalyst was studied. The amount of sodium hypophosphite added was 500 ppm, and the temperatures of SSP were 150 °C, 155 °C, and 160 °C. The time was 24 h, and the N_2_ flow rate was 30 mL/min. The effect of catalyst content on the relative viscosity of PA6/66 chips after solid-state polymerization is shown in Figure 1.

As shown in Figure 1, the relative viscosity of PA6/66 chips increases gradually with the increase in SSP temperature and time, which can be explained that the end group of the polymer can continue to react above the glass transition temperature of the polymer, and the molecular chain segment of the polymer continues to grow, resulting in a gradual increase in the relative viscosity of the polymer. Compared with the resins without a catalyst, the relative viscosity of the chips added with the catalyst increased faster under the same SSP conditions. This is because the phosphorus-containing catalyst can activate the carbonyl group, making the carbonyl carbon of the carboxyl group (-COOH) at the end of the polymer chain more electrophilic, which is more vulnerable to the nucleophilic attack of the amino group and promotes the polycondensation reaction of the carboxyl group (-COOH) and the amino group (-NH_2_) at the end of the chain. It can also be seen from Figure 1 that with the increase in solid-state polymerization reaction time, the increase in the viscosity of the sample without a catalyst decreased, while the relative viscosity of the sample with a catalyst increased further. The data points containing the relative viscosity of the catalyst chip at 150, 155, and 160 °C were linearly fitted, indicating that the relative viscosity increased linearly with time [8,27], and the corresponding slope was the viscosity-increasing rate, which was 0.056, 0.085, and 0.141 h^−1^, respectively. This phenomenon is consistent with the zero-order reaction kinetic equation, according to Equation (3):

(3) $$d\eta_{r}/dt=k*t$$

where *η_r_* represents relative viscosity, *t* represents the time of SSP, and *k* represents the rate of relative viscosity increase (h^−1^).

The activation energy of the reaction is calculated according to the Arrhenius Equation (4) [28]:

(4) lnk=lnA+Ea∗R∗(1/T)

where *k* represents the rate of relative viscosity increase (h^−1^), *E*a represents the activation energy of the SSP reaction (kJ/mol), *R* represents the Planck constant, and *T* represents the SSP temperature (K).

The activation energy of the SSP reaction with and without a catalyst is 141 kJ/mol and 182 kJ/mol, respectively. The lower activation energy may be related to diffusion control, and the higher value is usually considered to be related to chemical reaction control [8]. The SSP of PA6/66 copolymer conforms to the chemical reaction control model. The apparent activation energy of the reaction with a catalyst is significantly lower than that without a catalyst, indicating that the hypophosphite catalyst has a significant effect on reducing the activation energy of the end group reaction. The hydrolysis of hypophosphite under hydrothermal conditions produces acidic derivatives, which play an important role in the process of H^+^ carboxyl activation, terminal amino group (-NH_2_) nucleophilic attack, dehydration, and amide bond formation. At the same time, due to the small molecular size, the catalyst is easy to move, especially in the solid-state environment where the molecular chain segment activity is limited [29,30], thereby improving the reaction activity and reaction rate.

### 3.2. The Effect of Catalyst Content on SSP of PA6/66

In Section 3.1, it can be seen that the addition of a catalyst can effectively improve the SSP reaction rate. The effect of the catalyst content on the relative viscosity and SSP rate of the sample is shown in Table 1.

From Table 1, it can be seen that with the increase in catalyst addition, the viscosity and the SSP rate of the sample increase after SSP, which is due to the increase in catalyst addition, resulting in the increase in the number of catalytic active sites in the SSP. When the amount of catalyst added is more than 500 ppm, the increase in the viscosity-increasing rate of the sample is not further large, which indicates that the catalytic control concentration may exist in the SSP of PA6/66. Meanwhile, it was found that the content of P element in PA6/66 chips was very different from the theoretical feed content of P element in the polymerization reaction in Table 1, which showed that the catalyst was lost before SSP. At the same time, P element was detected in the extraction solution, indicating that the content of the active component of the catalyst in the polymer chips (measured by the content of P element in the chips) was greatly reduced, which was attributed to loss during the extraction process. Therefore, in the follow-up study, the catalytic efficiency of the solid-phase polymerization reaction was further evaluated based on the measured P element content of the chip.

**Figure 2:**
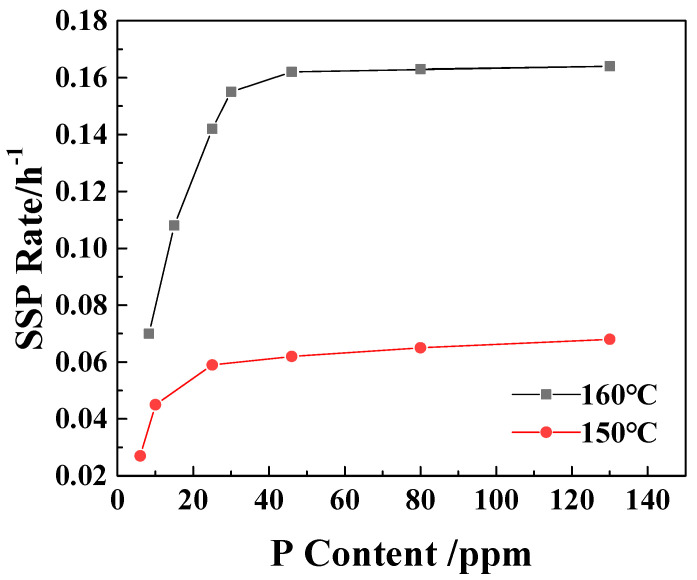
The effect of P content on the SSP rate of PA6/66 chips.

From Figure 2, it can be seen that with the increase in the P content in the sample, the SSP rate of the polymer increases. When the P element content is larger than 25 ppm at 150 °C and 30 ppm at 160 °C, the increase in the SSP rate of the sample is significantly reduced. This is consistent with the results of Table 1, which proves once again that the existence of catalytic control concentration and further increase in P content have no significant effect on the increase in viscosity-increasing rate.

Catalyst saturation exists in all catalytic reactions [31]. In the solid-state polymerization system, the number of chain end groups available for the reaction is limited, and their accessibility (i.e., in the amorphous region suitable for the reaction) is also limited. As the concentration of phosphorus increases, its physical presence in the amorphous region of the polymer may itself become an obstacle. The oxygen atom on phosphite (H_2_PO_2_^−1^) is a strong hydrogen bond acceptor. They will form new and possibly stronger hydrogen bonds with the amide hydrogen (-N-H) of the polyamide chain. Too many phosphorus species may overly anchor the polymer chain, forming dense and rigid hydrogen bond cross-linking points, thereby significantly reducing the chain mobility of the entire amorphous region. The decrease in chain mobility directly leads to the decrease in end-group diffusion rate, which inhibits the polymerization reaction [32].

### 3.3. The Effect of Catalyst Dispersion on the Relative Viscosity of PA6/66 Chips

The loss of catalyst occurs during the extraction process, which includes the catalyst diffusing from the chip to the extraction water and the polymer chip adsorbing the catalyst in the extraction. The surface catalyst of the chip is easier to diffuse into the water than the internal catalyst, which will cause the catalyst to be uniformly dispersed in the polymer. The catalyst content has a great influence on the viscosity and molecular weight of the polymer. In this section, the effect of the dispersion of the catalyst in the chips on the relative viscosity is studied.

In this paper, the polymer chip size is 2 mm in diameter and 2 mm in length. In this section, 0.5 mm surface samples are cut along the axial and cross-sectional directions of the chip. The surface polymer of multiple chips is defined as the outer layer, and the remaining is the inner layer. The viscosity of the chips was 4.34 after extraction and SSP. The outer and inner layers of the chip were taken to test the relative viscosity and P element content, as shown in Table 2. The viscosity of the outer and inner layers is 4.15 and 4.50, respectively. The relative viscosity of the inner layer is higher, and there is a significant difference in relative viscosity distribution. At the same time, there is also a significant difference in the content of P element. The catalyst content of the inner layer reaches 21.8 ppm, higher than that of the outer layer at 14.8 ppm. It can be seen that the uneven distribution of catalyst content between the outer and inner layers leads to the difference in relative viscosity. The viscosity-increasing rate of the inner layer is calculated to be 0.045 h^−1^, which is consistent with the results of the solid-state viscosity-increasing rate of the catalyst control concentration obtained in Section 3.2.

**Figure 3:**
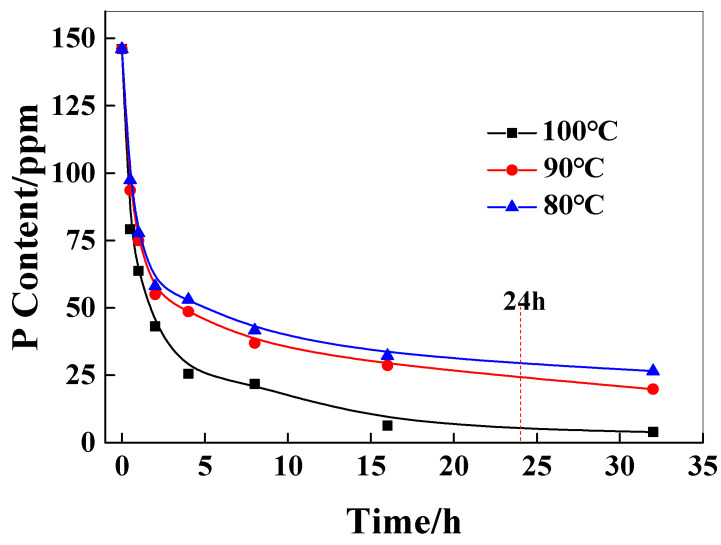
The effect of the extraction time on the P content in chips.

In practical applications of polymers, the relative viscosity difference in the polymer, due to the catalyst content of the inner and outer layers of chips, will significantly affect the molecular weight distribution of the polymer, resulting in poor performance uniformity of the subsequent products. Therefore, it is very important to control the catalyst content and distribution of the chip before the SSP process. As shown in Figure 3, it can be seen that with the increase in extraction time, the catalyst content in the chip decreases rapidly at first and then decreases gradually. The higher the extraction temperature, the greater the decrease in the content of the catalyst. This is because the higher the temperature, the higher the solubility of the catalyst in water and the faster the diffusion rate of the catalyst in the chip. When the extraction temperature is lower than 90 °C, the extraction time is less than 24 h, and the P content of the catalyst in the chip reaches 25 ppm, which reaches the control concentration of the catalyst.

Three samples with initial catalyst contents of 102 ppm, 146 ppm, and 219 ppm were used to investigate the residual amount of catalyst after 24 h extraction at 80 °C and 90 °C. The results are shown in Table 3. It was found that when the initial concentration of the chip is larger than 146 ppm, it can meet the requirement that the catalyst content is greater than or equal to 25 ppm. Consequently, the initial addition concentration of 146 ppm is appropriate.

**Figure 4:**
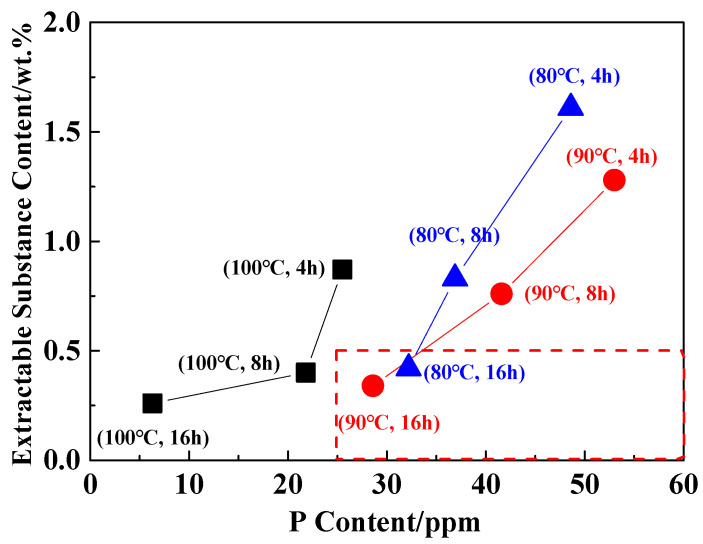
The content of the catalyst and extractable substances in the extraction process.

The extraction process of PA6/66 chips is mainly to remove the hot water extractable substances in order to improve the stability of subsequent polymer processing. Generally, the hot water extractable substances content is required to be less than 0.5 wt.% in subsequent applications [33]. In the above studies, it was found that the loss of the catalyst occurred simultaneously during the extraction process. Therefore, after the PA6/66 chip was extracted, the hot water extractable substances content was less than 0.5 wt.% under the premise that the catalyst content was higher than the control concentration. Figure 4 shows the content of extractable substances and P content under different extraction conditions. It can be seen that the extraction temperature is lower than 90 °C, the time is greater than 16 h, and the hot water extractable substances content of the chip is less than 0.5 wt.%. Table 4 shows the relative viscosity and extractable substances of the chip after optimizing the extraction process. The content of P element in the chip was 25 ppm, which was higher than the controlled catalytic concentration.

### 3.4. The Verification of Industrial Continuous Device

The author’s research institution has established a PA6/66 copolymer production line for manufacturing products with a 3.3 viscosity. The test line includes systems such as blending of raw materials and additives, polymerization, extraction, drying, solid-state polymerization, etc., which can achieve continuous production from raw materials to chips [34,35]. In the productive process, the sodium hypophosphite catalyst was mixed with the raw material in an aqueous solution and then put in the polymerization reactor. The catalyst concentration was 500 ppm (146 ppm by P element), the residence time of the chip in the extraction tower was 24 h, and the average temperature of the extraction tower was 90 °C. In order to prevent the adhesion of wet chips, the first-stage drying temperature was 135 °C, and the second-stage drying temperature was 155 °C. As shown in Table 4, the indexes of the chips after the optimized extraction process are as follows: the viscosity difference between the outer and inner layers is 0.12, and the extractable content is less than 0.41 wt.%, which can meet the requirements of the subsequent application.

## 4. Conclusions

Sodium hypophosphite served as a solid-state polymerization (SSP) catalyst for PA6/66 copolymerization, enabling controllable synthesis of high-viscosity PA6/66 resin with high catalytic activity. At 160 °C, the relative viscosity increase rate stably remained at 0.14 h^−1^, even as the polymer viscosity rose. The SSP kinetics at 150–160 °C conformed to a zero-order reaction, with an apparent activation energy of 141 kJ/mol. “Control concentrations” for the catalyst were identified as 25 ppm at 150 °C and 30 ppm at 160 °C, beyond which the viscosity increase rate no longer grew. Extraction at 80–90 °C for 16–24 h maintained catalyst concentrations in chip inner/outer layers above control levels, significantly reducing viscosity differences during efficient SSP, while extractable content stayed ≤0.41 wt.%. Sodium hypophosphite catalyst was applied to a 4500 tons/year continuous PA6/66 polymerization line via the extraction–drying–SSP process, stably producing uniform high-viscosity chips with extractable content <0.5 wt.%. It shortened viscosity-increasing time, reduced energy consumption, and resolved resin yellowing caused by prolonged high-temperature drying, offering a reliable technical solution for large-scale production of high-quality high-viscosity PA6/66 resin.

## Figures and Tables

**Table 1:** The effect of the catalyst content on the relative viscosity of PA6/66 chips.

Addition Content of Catalyst/ppm	P Theoretical Content of the Feed Material/ppm	P Content of the Resin/ppm ^①^	Relative Viscosity Before SSP	Relative Viscosity After SSP	SSP Rate/h^−1 ②^
0	0	0	2.71	3.57	0.036
250	73	12	2.63	3.69	0.044
350	102	17	2.70	3.82	0.047
500	146	25	2.65	4.76	0.088
750	219	53	2.70	4.85	0.090
1300	380	91	2.71	4.95	0.093

^①^ The content of P was obtained by the element content analysis. ^②^ SSP temperature: 155 °C, time: 24 h, and N_2_ flow rate: 30 mL/min.

**Table 2:** The viscosity of the inner and outer layers of the chip.

	Relative Viscosity		P Content
	Sample 1	Sample 2	
Outer layer	4.14	4.15	14.8
Inner layer	4.46	4.56	21.8

Sample 1 and Sample 2 are parallel tests.

**Table 3:** The change in catalyst content after extraction.

Sample	Extraction Temperature	
	80 °C	90 °C
1 (102 ppm)	26	23
2 (146 ppm)	32	25
3 (219 ppm)	36	27

**Table 4:** The index of chips after the optimized extraction process.

Sample	Relative Viscosity	Extractable Content/wt.%
Outer layer	3.95	0.37
Inner layer	4.07	0.41
Complete chip	4.01	0.39

## Data Availability

The original contributions presented in this study are included in the article. Further inquiries can be directed to the corresponding author.

## References

[1] Wang Z., Song M., Li X., Chen J., Liang T., Chen X., Yan Y. (2022). Copolymerization-regulated hydrogen bonds: A new routine for high-strength copolyamide 6/66 fibers. Polymers.

[2] Ehjeij D., Kopping J., Gabriel C., Wünsch J.R., Himmel H., Schröder R.R., Wilhelm M., Freudenberg J., Bunz U.H.F., Müllen K. (2025). Electrochemical exfoliation of graphene and formation of its copolyamide 6/66 nanocomposites by wet phase inversion and injection molding. Macromol. Chem. Phys..

[3] Vouyiouka S.N., Papaspyrides C.D. (2011). Solid-State Polymerization. Encyclopedia of Polymer Science and Technology.

[4] Zhang C., Wan L., Gu X., Feng L. (2015). A Study on a Prepolymerization Process of Aromatic-Contained Polyamide Copolymers PA(66-co-6T) via One-Step Polycondensation. Macromol. React. Eng..

[5] Fang Y., Wang L., Sun Y., Cheng K., Liu T., Wang X., Yu J., He Y. (2025). Direct solid state polymerization and structural properties of high heat resistant copolyamide PA56T. Acta. Polym. Sin..

[6] Xue B., Zhang Y., Zhang S., Fu P., Cui Z., Zhang Y., Li X., Pang X., Zhao W., Zhang X. (2025). Preparation and characterization of polyamide PA12T by direct solid state polymerization. Chem. Ind. Eng. Prog..

[7] Papaspyrides C.D., Vouyiouka S.N. (2009). Solid State Polymerization.

[8] Gaymans R.J., Amirtharaj J., Kamp H. (1982). Nylon 6 polymerization in the solid state. J. Appl. Polym. Sci..

[9] Zimmerman J., Kohan M.I. (2001). Nylon–selected topics. J. Polym. Sci. A Polym. Chem..

[10] Chen F.C., Griskey R.G., Beyer G.H. (1969). Thermally induced solid state polycondensation of nylon 66, nylon 6-10 and polyethylene terephthalate. AIChE J..

[11] Vouyiouka S., Karakatsani E., Papaspyrides C. (2005). Solid state polymerization. Prog. Polym. Sci..

[12] Papaspyrides C., Kampouris E. (1986). Influence of metal catalysts on solid state polyamidation of nylon salts. Polymer.

[13] Papaspyrides C.D. (1992). Solid State Polyamidation Processes. Polym. Inter..

[14] Katsikopoulos P.V., Papaspyrides C.D. (1994). Solid-state polyamidation of hexamethylenediammonium adipate. II. The influence of acid catalysts. J. Polym. Sci. A Polym. Chem..

[15] Liu P., Qu X., Jiang F., Zhang Z. (2017). Study on solid-state polycondensation and structural properties of nylon 6. New. Chem. Mater..

[16] Khripkov E.G., Kharitonov V.M., Kudryavtsev G.I. (1972). Some features of the polycondensation of hexamethylene diammonium adipinate. Fibre Chem..

[17] Papaspyrides C.D. (1987). Solid-state polyamidation of hexamethylenediammonium adipate in the presence of acid catalysts. I. Preparation of the catalyst containing monomer. J. Polym. Sci. C Polym. Lett..

[18] Srinivasan R., Almonacil C., Narayan S., Desai P., Abhiraman A.S. (1998). Mechanism, Kinetics and Potential Morphological Consequences of Solid-State Polymerization. Macromolecules.

[19] Li L.F., Huang N.X., Tang Z.L., Hagen R. (2001). Reaction Kinetics and Simulation for the Solid-State Polycondensation of Nylon 6. Macromol. Theory Simul..

[20] Zhang R., Dong J., Zang Y. (1989). Solid-Phase Polymerization of Nylon-6 and Its Properties. J. Beijing Inst. Fash.Technol..

[21] Kaushik A., Gupta S.K. (1992). A molecular model for solid-state polymerization of nylon 6. J. Appl. Polym. Sci..

[22] Kulkarni M.R., Gupta S.K. (1994). Molecular Model for Solid-state Polymerization of Nylon 6. II. An Improved Model. J. Appl. Polym. Sci..

[23] Vouyiouka S.N., Papaspyrides C.D., Weber J., Marks D. (2005). Solid State Polymerization: Evaluation of Pertinent Kinetic Models. J. Appl. Polym. Sci..

[24] (2019). Test Methods for Fiber-Grade Polycaprolactamide (PA6) Chips.

[25] Zheng W., Mcauley K.B., Marchildon E.K., Yao K.Z. (2007). Melt-phase nylon 612 polycondensation kinetics: Effects of sodium hypophosphite catalyst. Can. J. Chem. Eng..

[26] Lysek B.A., Ables R.W. (1999). Nucleation of Polyamides in the Resence of Hypophosphite. U.S. Patent.

[27] Fujimoto A., Mori T., Hiruta S. (1988). Solid polymerization of Nylon 66. Nippon Kagaku Kaishi.

[28] Abedi A., Bahrami S.H. (2006). Investigation on solid-state polymerisation reaction mechanism of nylon-6. J. Chem. Res..

[29] Papaspyrides C.D., Vouyiouka S.N. Mechanistic Aspects of Solid State Polyamidation Processes. Proceedings of the 7th MoDeSt Conference.

[30] Vouyiouka S.N., Papaspyrides C.D., Pfaendner R. (2006). Catalyzed solid-state polyamidation. Macromol. Mater. Eng..

[31] Wursthorn L., Beckett K., Rothbaum J.O., Cywar R.M., Lincoln C., Kratish Y., Marks T.J. (2023). Selective lanthanide-organic catalyzed depolymerization of nylon-6 to ϵ-caprolactam. Angew. Chem. Int. Ed..

[32] Chen S., Zhong S., Chen W. (2024). Kinetics Comparison of Solid-State Polycondensation and Melt Postpolycondensation for Polyamide 6 with Controllable Extractive Content. Ind. Eng. Chem. Res..

[33] (2011). Fiber Grade Polycaprolactam Chip.

[34] Jiang F., Yu C.X., Li X., Qiu Z.C., Huang Y.M., Hu Z.Y., Wang X. (2021). Method for Preparing Copolyamide and Copolyamide. China Patent.

[35] Jiang F., Yu C.X., Li X., Hu Z.Y., Luo X., He Y.Y., Zhao L. (2021). A Method for Increasing the Molecular Weight of Copolyamide and Its Product. China Patent.

